# Characterization of Cardiac, Vascular, and Metabolic Changes in Young Childhood Cancer Survivors

**DOI:** 10.3389/fped.2021.764679

**Published:** 2021-12-08

**Authors:** Olof Broberg, Ingrid Øra, Thomas Wiebe, Constance G. Weismann, Petru Liuba

**Affiliations:** ^1^Pediatric Heart Center, Skåne University Hospital, Lund, Sweden; ^2^Clinical Sciences, Department of Pediatrics, Lund University, Lund, Sweden; ^3^Pediatric Oncology, Skåne University Hospital, Lund, Sweden

**Keywords:** childhood cancer, cardiac function, carotid artery, reactive hyperaemia index, apolipoprotein

## Abstract

**Background:** Childhood cancer survivors (CCS) are at an increased risk for cardiovascular diseases (CVD). It was the primary aim of this study to determine different measures of cardiac, carotid, lipid, and apolipoprotein status in young adult CCS and in healthy controls.

**Methods:** Cardiac and common carotid artery (CCA) structure and function were measured by ultrasonography. Lipids and apolipoproteins were measured in the blood. Peripheral arterial endothelial vasomotor function was assessed by measuring digital reactive hyperemia index (PAT-RHI) using the Endo-PAT 2000.

**Results:** Fifty-three CCS (20–30 years, 35 men) and 53 sex-matched controls were studied. The CCS cohort was divided by the median dose of anthracyclines into a low anthracycline dose (LAD) group (50–197 mg/m^2^, *n* = 26) and a high anthracycline dose (HAD) group (200–486 mg/m^2^, *n* = 27). Carotid distensibility index (DI) and endothelial function determined by PAT-RHI were both lower in the CCS groups compared with controls (*p* < 0.05 and *p* = 0.02). There was no difference in carotid intima media thickness. Atherogenic apolipoprotein-B (Apo-B) and the ratio between Apo-B and Apoliprotein-A1 (Apo-A1) were higher in the HAD group compared with controls (*p* < 0.01). Apo-B/Apo-A1-ratio was over reference limit in 29.6% of the HAD group, in 15.4% of LAD group, and in 7.5% of controls (*p* = 0.03). Measured lipid markers (low density lipoprotein and total cholesterol and triglycerides) were higher in both CCS groups compared with controls (*p* < 0.05). Systolic and diastolic function were measurably decreased in the HAD group, as evidenced by lower EF (*p* < 0.001) and lower é-wave (*p* < 0.005) compared with controls. CCA DI correlated with Apo-B/Apo-A1-ratio and Apo-A1. Follow-up time after treatment correlated with decreased left ventricular ejection fraction (*p* = 0.001).

**Conclusion:** Young asymptomatic CCS exhibit cardiac, vascular, lipid, and apolipoprotein changes that could account for increased risk for CVD later in life. These findings emphasize the importance of cardiometabolic monitoring even in young CCS.

## Introduction

Cardiovascular disease (CVD) including cardiomyopathy, ischemic heart disease, and cerebrovascular disease amount to the most prevalent non-cancerous causes of death in childhood cancer survivors (CCS) (1, 2). The risk for CVD in CCS is at least in part attributed to previous treatment regimens with cardiotoxic anthracyclines (AC) and radiotherapy (3). Recent studies have shown that CCS have increased carotid intima media thickness (CIMT), stiffer arteries, and endothelial dysfunction (4–8). These deviations are now recognized as markers of early atherosclerosis and predict later onset of ventricular dysfunction, stroke, and ischaemic heart disease (9–13). CCS are also at increased risk for the metabolic syndrome, dyslipidaemia, obesity, and hormonal abnormalities (14), which could further contribute to their multifold risk for cardiovascular morbidity and mortality (15).

Previous studies have largely focused on either cardiac or vascular abnormalities, mostly in patients during or soon after treatment or in older CCS. Assessing the early onset of both vascular and cardiac changes may provide more accurate insights with regard to the development of future CVD in CCS, leading to better preventive strategies. Therefore, the aim of this work was to determine different cardiac, carotid, lipid, and apolipoprotein markers for CVD in young Swedish CCS and the associations between these markers. To evaluate this, we performed a cross-sectional study on young CCS treated with AC, without any previous or current overt CVD, and compared them with age- and sex-matched healthy controls.

## Methods and Study Population

We conducted an observational cross-sectional case–control study of cardiovascular status in young adults who survived cancer during childhood after treatment at the Department of Pediatric Oncology of the Skåne University Hospital Lund, Sweden. All data collection of CCS and controls was performed from April 2014 to April 2017. Inclusion criteria were: cancer diagnosis under the age of 18, treatment with AC among other chemotherapeutic agents, with and without radiotherapy, survival more than 5 years after disease remission, and age 20–30 years at inclusion. Exclusion criteria were: brain tumor diagnosis (these patients are known to have endocrine disorders and motor deficits), previous overt CVD or any cardiovascular complication during cancer treatment, any chronic disease or syndrome, or pregnancy. Informed written consent was obtained from all study participants. The study was approved by the Regional Ethical Committee for Human Research, Lund, Sweden (DNR 2013/205).

Childhood cancer survivors were identified in the population based BORISS registry (16) for childhood malignancies in southern Sweden. One-hundred-fifty-two CCS met the eligibility criteria and received a written invitation to participate. If no answer was received an additional invitation was sent. An equal number of healthy controls with similar sex and age distribution were recruited by written announcements at the Skåne University Hospital area in Lund, Sweden, and these were examined exactly in the same way and during the same time-period as the CCS.

To evaluate vascular and cardiac effects of previous treatment with AC, CCS were divided into two subgroups based on the median cumulative dose of AC: low cumulative AC-dose (LAD) group and high cumulative dose (HAD) group. AC-doses were converted to doxorubicin equivalents using conversion factors: 0.83 for duanorubicine, 0.67 for epirubicine, 5.00 for idarubicine, and 4.00 for mitoxantrone (17). Age at diagnosis and follow-up time were retrieved from the registry.

All study participants completed a questionnaire (18) previously used in our institution regarding current regular use of medications (cardiovascular medicines, statins), tobacco use (type (cigarettes or smokeless tobacco), dose and frequency), level of physical exercise (sports or gym training, hours/week). Systolic and diastolic brachial blood pressure were measured after 15 min of rest in supine position in the right arm using a calibrated wall-hung aneroid sphygmomanometer at the same time as carotid ultrasonography was performed. Weight and height were measured (using a calibrated scale and a stadiometer). Overweight was defined as BMI ≥ 25 and <30 kg/m^2^ and obesity was defined as BMI ≥ 30 kg/m^2^.

### Laboratory Analyses

Fasting blood samples were collected to analyze lipid and apolipoproteinmarkers for CVD in plasma and serumas follows; triglycerides (TG, ref. 0.45–2.60 mM), low-density lipoprotein (LDL, ref. 1.20–4.30 mM), high-density lipoprotein (HDL, ref. women 1.00–2.70 mM, men 0.80–2.10 mM), total cholesterol (ref. 2.90–6.10 mM), creatinine (ref. women 45–90μM, men 60-105μM), apolipoprotein A1 (Apo-A1, ref. women 1.08–2.25 g/L, men 1.04–2.02 g/L), and apolipoprotein B (Apo-B, ref. women 0.60–1.17g/L, men 0.66–1.33g/L). The Apo-B/Apo-A1 ratio was calculated (ref. <0.90 for men and <0.80 for women). Estimated glomerular filtration rate (eGFR, ref. 80–125mL/min/1.73 m^2^) was estimated from creatinine and cystatine-C (19). All the blood samples were collected and analyzed by standard assays (Roche Diagnostics, Basel, Switzerland). All reference values above are age and sex-specific and were provided by the department of Clinical Chemistry at the Skåne University Hospital.

### Echocardiography

Electrocardiography-gated carotid ultrasonography and echocardiography were performed by a single investigator (OB) according to a standardized protocol, and image acquisition was done according to the American Society of Echocardiography guidelines (20, 21). An echocardiograph (EPIQ-7, Philips Medical Systems, Andover, Massachusetts, USA) equipped with an X5-1 probe (1–5 MHz) was used for echocardiography, and a L15-7io (7–15 MHz) linear array transducer for vascular ultrasound was used for carotid ultrasound. The investigator who performed these assessments was blinded to the status of the study participants. All cardiac and carotid measurements were performed offline (QLAB, Philips Healthcare Netherlands) and were averaged over three cardiac cycles.

M-mode measurements of the septal thickness (IVS) and left ventricular posterior wall thickness (LVPW) along with left ventricular internal diameters (LVID) in systole and diastole were obtained in the parasternal view to evaluate cardiac morphology. Left ventricular mass was calculated based on the M-mode measures using the Devereux formula (21). Shortening fraction (SF) was calculated using the M-mode measures. Left ventricular ejection fraction (LVEF) was calculated using the Simpson biplane method using the apical two- and four-chamber views. Mitral valve E- and A-wave were obtained from the apical four-chamber view. To determine the left ventricular ś and é-wave, tissue-Doppler was recorded at mitral annulus septal and lateral wall point (21).

Loops of three to five beats of both right and left common carotid arteries (CCA) in the longitudinal view were obtained using electrocardiography-gated ultrasonography. The far wall of a 1-cm long segment of the proximal CCA was used to measure intima media thickness (CIMT) with a semiautomated tracing algorithm (QLAB, Philips Healthcare Netherlands). The CCA diastolic diameter (CCA Dd) and systolic diameter (CCA Ds) were measured from the leading edge of the near wall (adventia/media border) to the leading edge of the far wall. The distensibility index (DI) and the β-stiffness index (SI) were calculated using previously described methods (22, 23):


$$\begin{array}{l} DI\;\left(\frac{\%\;diameter\;change}{10mmHg}\right)=\frac{Ds-Dd}{Dd^{*}\left[SBP-DBP\right]}*1000 \end{array}$$

$$\begin{array}{l} SI\;\left(no\;unit\right)=\ln\left(\frac{SBP}{DBP}\right)/\left(\frac{\left[Ds-Dd\right]}{Dd}\right) \end{array}$$

Inter- and intraobserver variability for the ultrasound measurements were assessed in a subgroup of 23 participants. Interobserver and intraobserver intraclass correlation coefficient (ICC) for all carotid, doppler, and M-mode measurements were >0.9. Interobserver ICC for LVEF was 0.84 and 0.90 for intraobserver ICC.

### Peripheral Arterial Tonometry–Reactive Hyperaemia Index

Peripheral vascular endothelial function was assessed by measuring peripheral arterial tonometry–reactive hyperaemia index (PAT-RHI) using the Endo-PAT 2000 (Itamar Medical, Caesarea, Israel). This method has previously been reported to correlate with endothelial function in the coronary circulation (24) and to be predictive for CVD (25). All measurements were done following a standardized procedure specified by the manufacturer (www.itamar-medical.com). After 5 min of tracing bilateral baseline index-finger oscillations of blood flow, a blood pressure cuff was inflated to occlude blood flow into the non-dominant arm for 5 min. Following pressure release, tracings were obtained for another 5 min. A computerized software with a proprietary algorithm automatically calculated the reactive hyperaemia index from the fold increase in the pulse wave amplitude relative to baseline. The reference limit for PAT-RHI was set at >1.67, as specified by the manufacturer.

### Statistical Analyses

Statistical analyses were performed with SPSS software (IBM, version 27; SPSS, Chicago, Illinois, USA). Results were reported as means (adjusted and unadjusted) and standard deviations (SD) or numbers (proportions and percentages). Based on the histogram inspection, summary statistics of the software (providing information on means and standard deviations and on medians and quantiles), and given skewness >-1 but <1, we assumed variables to be approximately normally distributed.

For baseline characteristics, continuous variables were compared between groups using ANOVA and Student *T*-test as appropriate. For dichotomous variables the Chi-square test was used. For group comparisons of outcome variables (DI/SI, CIMT, PAT-RHI, cardiac diastolic and systolic function, and lipids and apolipoproteins) ANCOVA was used. Unbalanced baseline variables were adjusted for. Blood pressure was adjusted for height. In a subgroup analysis adjusting for treatment variables (follow-up time after treatment completion, cranial and mediastinal radiotherapy) only the LAD and the HAD groups were compared. For carotid measures, CCS treated with cranial radiotherapy was compared with CCS without this treatment and results were adjusted for the cumulative AC dose. *Post-hoc* tests were performed using the Bonferroni method for three-group comparisons and LSD for subgroup analysis of only CCS. Partial correlations adjusted for age and sex were performed within the CCS group. For ANCOVA, means and adjusted means were reported. A two-sided *p*-value below 0.05 was considered statistically significant.

## Results

### Characteristics of the Study Participants

Description of the study cohort including distribution of cancer diagnosis is shown in Table 1. Fifty-three CCS and an equal number of controls (mean age 25.3 ± 2.4 and 24 ± 2.4 years, respectively), were studied. The median AC dose of CCS was 197.0 mg/m^2^. As described in the Methods, based by the median AC dose, the CCS cohort was divided into two groups: low AC dose (LAD; *n* = 26) and high AC dose (HAD; *n* = 27). Among CCS, leukemia was the most common cancer diagnosis (*n* = 23 of whom three had been treated for acute myeloid leukemia and 20 for acute lymphoblastic leukemia). Four CCS had been treated for rhabdomyosarcoma, one for Ewing's sarcoma, one for osteosarcoma, five for Wilms's tumor, 11 for Hodgkin's disease, and eight for non-Hodgkin's lymphoma.

**Table 1:** Characteristics of the study cohort divided in subjects with low (LAD) or high (HAD) cumulative dose of anthracyclines.

**Variables**	**Controls (*n* = 53)**	**LAD (*n* = 26)**	**HAD (*n* = 27)**
Age, years (SD)	24.4 (2.4)	25.0 (2.4)	25.6 (2.5)
Male sex, n	35 (66.0%)	15 (57.7%)	17 (63.0%)
Height, cm (SD)	179.1 (8.7)	178.0 (10.1)	171.8 (9.8)^**^
Weight, kg (SD)	79.2 (15.3)	78.2 (14.4)	71.6 (13.3)
BMI, kg/m^2^ (SD)	24.60 (3.79)	24.64 (3.53)	24.17 (3.58)
Overweight, n	16 (30.0%)	11 (42.0%)	9 (33.3%)
Obesity, n	5 (9.4 %)	2 (7.8%)	2 (7.4%)
SBP, mmHg, (SD)	118.8 (11.6)	118.2 (10.1)	119.1 (12.4)
DBP, mmHg, (SD)	73.4 (5.9)	75.7 (8.3)	76.3 (8.8)
Hypertension, n	1 (1.9%)	2 (7.7%)	2 (7.4%)
Resting HR, beats/min, (SD)	66.2 (11.3)	70.0 (11.2)	74.1 (11.0)^*^
Exercise (hours/week), (SD)	4.4 (2.9)	4.6 (6.5)	4.2 (2.2)
Tobacco users, n	9 (17.0%)	6 (23.1%)	6 (22.2%)
Regular smokers, n	2 (3.8%)	1 (3.8%)	2 (7.4%)
GFR, ml/min/1,73m^2^ (SD)	94.98 (9.00)	92.41 (12.05)	93.88 (12.47)
**Cancer Diagnosis**	
Leukaemia, n		5 (19.2%)	18 (66.7%)
Hodgkin lymphoma, n		10 (38.5%)	1 (3.70%)
Non-Hodgkin lymphoma, n		7 (26.9%)	1 (3.70%)
Sarcoma, n		0	6 (22.2%)
Wilms tumor, n		4 (15.4%)	1 (3.7%)
Age at diagnosis, y (SD)		10.8 (5.6)	6.0 (4.5)^*^
Follow-up time, y (SD)		13.4 (5.4)	18.0 (5.2)^*^
Cumulative AC, mg/m^2^ (SD)		143.6 (33.1)	277.3 (84.9)^*^
Radiotherapy, n		11 (42.3%)	13 (48.1%)
Mediastinal radiotherapy, n		8 (30.8%)	2 (7.4%)
Cranial radiotherapy, n		0	8 (29.6%)
Other radiotherapy, n		3 (11.5%)	3 (11.1%)
Cumulative dose radiotherapy, Gy (SD)		22.5 (7.4)	26.2 (11.0)

*Values are presented as mean and SD or as number and %. p-values were calculated by one-way analysis of variance (ANOVA); intergroup differences were calculated by the Bonferroni post-hoc test with correction for multiple comparisons, standard T-test or with chi-square test*:

* *p <0.05 vs controls or LAD vs HAD group*;

** *p <0.005 vs controls. LAD, low anthracycline dose; HAD, low anthracycline dose**;** BSA, body surface area; BMI, body mass index; SBP, systolic blood pressure; DBP, diastolic blood pressure; HR, heart rate, AC, anthracycline*.

In addition to AC, 24 CCS (11 in the LAD group and 13 in the HAD group) had radiotherapy as follows: mediastinal radiotherapy in 10 CCS (all with lymphoma, of whom eight were in the LAD group and two in the HAD group), cranial radiotherapy in eight (all with leukemia in the HAD group), and radiotherapy to other organs in six (three in each group). The mean follow-up time after the end of cancer treatment in the CCS cohort was 15.8 (± 5.8) years. The LAD group was older at the time of diagnosis and had a shorter follow-up compared with the HAD group (*p* = 0.001 and *p* = 0.003, respectively).

The HAD group was shorter compared with the control group (*p* = 0.004) and had a higher resting heart rate (*p* = 0.010). Blood pressure was similar in CCS compared with controls. There were five users of smokeless tobacco in the control group and five and four in the LAD and HAD groups, respectively. The control group had four cigarette users (two daily users and two occasional or previous users). The LAD-group had one daily user and the HAD group had two occasional or previous users. There was no difference in tobacco use between controls and CCS groups. None of the participants were on cardiovascular medications. Exercise (hours/week) was similar between controls and CCS.

### Apolipoproteins and Lipid Biomarkers

Results of group comparisons between controls and CCS groups of lipid biomarkers and apolipoproteins are outlined in Table 2. Compared with controls, markers for atherosclerosis, Apo-B and the Apo-B/Apo-A1-ratio, were higher in the HAD group (*p* = 0.006 and *p* = 0.011, respectively). Apo-B/Apo-A1 ratio exceeded the upper reference range for age and sex in 7.5% of controls compared with 15.4% in the LAD group and 29.6% in the HAD group (*p* = 0.030). Apo-A1 and HDL were similar between controls and CCS groups. Compared with controls LDL, total cholesterol, and TG were higher in the HAD group (*p* < 0.005). Following adjustment for radiotherapy and follow-up time, cholesterol and Apo-B were higher in the HAD group when compared to the LAD group (*p* = 0.040 and *p* = 0.041, respectively).

**Table 2:** Different biomarkers for lipid status in childhood cancer survivors with low (LAD) or high (HAD) cumulative dose of anthracyclines.

**Variables**	**Controls**	**LAD (*****n*** **=** **26)**		**HAD (*****n*** **=** **27)**	
		**Subgroup analysis^a^**		**Subgroup analysis^a^**	
LDL (mM)	2.22 (0.77)	2.64 (0.69)^*^		3.06 (0.77)^**^	
Adjusted Means	2.25 (0.77)	2.63 (0.74)	*2.61 (0.83)*	2.69 (0.78)	*3.05 (0.83)*
HDL (mM)	1.40 (0.32)	1.36 (0.30)		1.29 (0.34)	
Adjusted Means	1.41 (0.33)	1.37 (0.32)	*1.34 (0.36)*	1.28 (0.34)	*1.33 (0.36)*
TG (mM)	0.79 (0.35)	1.01 (0.58)^*^		1.37 (0.93)^**^	
Adjusted Means	0.81 (0.58)	1.00 (0.59)	*1.04 (0.85)*	1.33 (0.62)	*1.32 (0.85)*
Cholesterol^b^ (mM)	3.84 (0.82)	4.32 (0.76)^*^		4.82 (0.88)^**^	
Adjusted Means	3.89 (0.83)	4.31 (0.78)	*4.29 (0.90)*	4.71 (0.85)	*4.85(0.92)^b^*
Apo-A1 (g/L)	1.42 (0.22)	1.57 (0.40)		1.44 (0.27)	
Adjusted Means	1.44 (0.24)	1.45 (0.23)	*1.44 (0.28)*	1.43 (0.24)	*1.48 (0.29)*
Apo-B (g/L)	0.72 (0.20)	0.80 (0.19)		0.92 (0.21)^**^	
Adjusted Means	0.73 (0.20)	0.80 (0.19)	*0.79 (0.22)*	0.90 (0.20)	*0.93 (0.21)^b^*
Apo-B/Apo-A1 ratio	0.52 (0.16)	0.56 (0.14)		0.65 (0.19)^**^	
Adjusted Means	0.52 (0.17)	0.56 (0.16)	*0.56 (0.19)*	0.65 (0.17)	*0.65 (0.18)*

*LDL, low-density lipoprotein; HDL, high-density lipoprotein; TG, triglycerides; Apo, apolipoprotein. Values are shown as mean (SD). Differences were calculated by one-way analysis of variance adjusting for unbalanced baseline variables (ANCOVA) and intergroup differences were calculated by the Bonferroni post-hoc test with correction for multiple comparisons*:

* *p <0.05 vs. controls*;

** *p <0.005 vs. controls*;

a *Subgroup analysis LAD vs. HAD group adjusted for follow-up time and radiotherapy—adjusted means (SD)*,

b *p <0.05 vs. LAD group ^b^1 sample result for cholesterol missing in the HAD group. Italic values are subgroup analysis*.

In order to exclude a possible confounding effect of overweight and obesity on Apo-B and the Apo-B/Apo-A1 ratio, a separate analysis of CCS (*n* = 29) and controls (*n* = 32) with a normal weight (BMI <25 kg/m^2^) was done: CCS had higher Apo-B of 0.85, 95% CI [0.78–0.92] vs 0.69, 95% CI [0.62–0.76], *p* = 0.001 and Apo-B/Apo-A1-ratio of 0.58, 95% CI [0.52–0.64] vs 0.48, 95% CI [0.42–0.54], *p* = 0.016.

### Carotid Measurements and PAT-RHI

These data are outlined in Table 3. CIMT was not different between controls and CCS groups (*p* = 0.90). The mean DI of the right and left CCA was lower in both LAD and HAD groups compared with controls (*p* < 0.001 and *p* = 0.018, respectively). The mean SI of the right and left CCA was higher in the LAD and HAD groups compared with controls (*p* = 0.041 and *p* < 0.001, respectively). Further, mean DI was lower, and mean SI was higher in the HAD group compared with the LAD group (*p* = 0.040 and *p* = 0.002, respectively). After adjusting for follow-up time and cranial and mediastinal radiotherapy, the HAD group displayed lower DI and higher SI compared to the LAD group (*p* = 0.007 and *p* = 0.020, respectively).

**Table 3:** Vascular outcomes in controls and childhood cancer survivors with low (LAD) or high (HAD) cumulative dose of anthracyclines.

**Variables**	**Controls (*n* = 53)**	**LAD (*****n*** **=** **26)**		**HAD (*****n*** **=** **27)**	
		**Subgroup analysis^a^**		**Subgroup analysis^a^**	
PAT-RHI^‡^	2.23 (0.61)	1.86 (0.69)^*^		2.02 (0.62)	
Adjusted Means	2.20 (0.66)	1.85 (0.64)	*1.88 (0.73)*	2.09 (0.66)	*1.99 (0.72)*
**CCA measurements**
CIMT right CCA (mm)	0.448 (0.052)	0.454 (0.036)		0.460 (0.076)	
Adjusted Means	0.447 (0.058)	0.451 (0.057)	*0.455 (0.065)*	0.462 (0.058)	*0.456 (0.066)*
CIMT left CCA (mm)	0.439 (0.055)	0.452 (0.041)		0.455 (0.064)	
Adjusted Means	0.439 (0.058)	0.451 (0.058)	*0.458 (0.062)*	0.457 (0.058)	*0.450 (0.061*)
CIMT mean left/right CCA (mm)	0.444 (0.047)	0.453 (0.036)		0.458 (0.069)	
Adjusted Means	0.444 (0.051)	0.451 (0.051)	*0.455 (0.062)*	0.458 (0.051)	*0.453 (0.061)*
DI right CCA (%/10 mmHg)	3.43 (0.96)	2.93 (0.76)		2.29 (0.64)^**^	
Adjusted Means	3.40 (0.88)	2.92 (0.87)	*2.88 (0.76)*	2.23 (0.88)	*2.29 (0.75)^b^*
DI left CCA (%/10 mmHg)	3.51 (0.80)	2.96 (0.59)^**^		2.50 (0.69)^***^	
Adjusted Means	3.54 (0.77)	2.97 (0.76)	*2.91 (0.75)*	2.43 (0.76)	*2.54 (0.74)*
Mean DI (%/10 mmHg)	3.47 (0.81)	2.94 (0.60)^**^		2.40 (0.63)^***^	
Adjusted Means	3.49 (0.76)	2.97 (0.75)	*2.90 (0.70)*	2.32 (0.75)	*2.44 (0.69)^b^*
SI right CCA (β)	3.31 (0.77)	3.84 (1.10)		4.95 (1.40)^***^	
Adjusted Means	3.35 (1.11)	3.87 (1.11)	*3.94 (1.40)*	5.01 (1.12)	*4.94 (1.39)^b^*
SI left CCA (β)	3.31 (0.93)	4.02 (0.63)^*^		4.92 (1.40)^***^	
Adjusted means	3.30 (1.08)	4.00 (1.07)	*4.19 (1.29)*	4.94 (1.09)	*4.76 (1.28)*
Mean SI (β)	3.31 (0.78)	3.93 (0.76)^*^		4.94 (1.30)^****^	
Adjusted means	3.28 (0.99)	3.91 (0.98)	*4.06 (1.26)*	5.01 (0.98)	*4.82 (1.28)^b^*

*PAT-RHI, reactive hyperaemia index; CCA, common carotid artery; CIMT, carotid intima media thickness; DI, distensibility index; SI, stiffness index. Values are shown as mean (SD). Group differences were calculated by one-way analysis of covariance adjusting for heart rate and height (ANCOVA). Intergroup differences were calculated by the Bonferroni post-hoc test with correction for multiple comparisons*;

* *p <0.05 vs. control*,

** *p <0.005 vs. control*,

*** *p <0.05 vs. LAD group and p <0.005 vs. controls*,

**** *p <0.005 vs. controls and LAD group*,

a *Subgroup analysis LAD vs. HAD group adjusted for follow-up time and radiotherapy—adjusted means (SD)*,

b *p <0.05 vs. LAD group*.

‡ *, missing data for 1 control and 2 in the LAD group. Italic values are subgroup analysis*.

When comparing CCS with and without cranial radiotherapy (Table 4), SI of the left CCA was higher in those with cranial radiotherapy (*p* = 0.011) after adjusting for the total AC dose.

**Table 4:** Carotid measures in CCS treated with cranial radiotherapy.

**Variables**	**CCS (*N* = 45)**	**Cranial RT (*N* = 8)**	***P-*value**
CIMT right CCA (mm)	0.455 (0.063)	0.455 (0.034)	0.960
Adjusted means	0.457 (0.057)	0.448 (0.058)	0.696
CIMT left CCA (mm)	0.455 (0.057)	0.445 (0.035)	0.617
Adjusted means	0.457 (0.051)	0.437 (0.054)	0.331
CIMT mean left/right CCA (mm)	0.454 (0.058)	0.450 (0.026)	0.813
Adjusted means	0.456 (0.051)	0.442 (0.054)	0.499
DI right CCA (%/10 mmHg)	2.66 (0.78)	2.11 (0.45)	0.060
Adjusted Means	2.64 (0.71)	2.19 (0.74)	0.123
DI left CCA (%/10 mmHg)	2.82 (0.66)	2.24 (0.60)	0.027
Adjusted means	2.80 (0.64)	2.32 (0.65)	0.060
Mean DI (%/10 mmHg)	2.75 (0.66)	2.18 (0.51)	0.023
Adjusted means	2.74 (0.63)	2.25 (0.63)	0.054
SI right CCA (β)	4.31 (1.34)	5.22 (1.21)	0.81
Adjusted means	4.30 (1.28)	5.08 (1.33)	0.159
SI left CCA (β)	4.29 (0.86)	5.51 (1.96)	0.006
Adjusted means	4.29 (1.11)	5.47 (1.13)	**0.011**
Mean SI (β)	4.27 (1.02)	5.36 (1.55)	0.121
Adjusted means	4.34 (1.11)	5.27 (1.13)	0.099

*CCA, common carotid artery; CIMT, carotid intima media thickness; DI, distensibility index; SI, stiffness index; RT, radiotherapy. Values are shown as mean (SD). Group differences were calculated by one-way analysis of covariance adjusting for mean anthracycline dose (ANCOVA)*.

Peripheral arterial tonometry–reactive hyperaemia index was significantly lower in the whole CCS cohort compared with controls (CCS; 1.94, 95% CI 1.77–2.12, controls; 2.23, 95% CI 2.06–2.40, *p* = 0.013). Likewise, the proportion of study participants with an abnormal PAT-RHI defined as <1.67 was significantly higher in the CCS cohort when compared with the controls (*n* = 19 of CCS vs. nine of controls*, p* = 0.046). The LAD group, but not the HAD group, had lower PAT-RHI compared with controls (*p* = 0.017) and there was no difference between the LAD and the HAD group (*p* = 0.36), as shown in Table 3. In the subgroup analysis, controlling for follow-up time and radiotherapy, comparing the LAD and the HAD groups, there was no difference between the groups for PAT-RHI (*p* = 0.47).

### Echocardiography

Group comparisons are outlined in Table 5. Both CCS groups had lower cardiac mass compared with controls (*p* = 0.011 and *p* = 0.041, respectively). Compared with controls and the LAD group, systolic LVID was larger in the HAD group (*p* = 0.001 and *p* = 0.014, respectively). Systolic and diastolic LVPW were smaller in both the CCS groups when compared with controls (*p* < 0.05 for diastolic LVPW and *p* < 0.005 for diastolic LVPW). LV systolic function (LVEF) was lower in the HAD group compared with controls and the LAD group (*p* < 0.001 and *p* = 0.020, respectively). Six CCS in the HAD group had LVEF <55% (49–54.2%) compared with none in the LAD group and one among controls (LVEF 54.9%).

**Table 5:** Cardiac outcomes in controls and childhood cancer survivors with low (LAD) or high (HAD) cumulative dose of anthracyclines.

**Variables**	**Controls (*N* = 53)**	**LAD (*****N*** **=** **26)**		**HAD (*****N*** **=** **27)**	
		**Subgroup analysis^a^**		**Subgroup analysis^a^**	
**M-mode Left Ventricle**
IVSd/BSA (mm/m^2^)	4.15 (0.78)	3.90 (0.88)		4.04 (0.95)	
Adjusted means	4.14 (0.84)	3.97 (0.81)	*4.07 (0.96)*	4.01 (0.86)	*3.88 (0.96)*
LVIDd/BSA (mm/m^2^)	26.65 (3.35)	26.67 (2.90)		27.78 (4.42)	
Adjusted means	26.74 (3.39)	26.86 (3.23)	*26.57 (4.02)*	27.46 (3.34)	*27.88 (4.03)*
LVPWd/BSA (mm/m^2^)	4.30 (1.00)	3.74 (0.66)^*^		3.75 (0.71)^*^	
Adjusted means	4.23 (0.86)	3.77 (0.82)	*3.77 (0.68*)	3.73 (0.87)	*3.73 (0.68)*
IVSs/BSA (mm/m^2^)	5.94 (1.05)	5.63 (1.18)		5.77 (1.42)	
Adjusted Means	5.96 (1.18)	5.68 (1.14)	*5.83 (1.38)*	5.69 (1.20)	*5.58 (1.38)*
LVIDs/BSA (mm/m^2^)	16.24 (2.49)	16.47 (2.29)		18.25 (3.44)^***^	
Adjusted Means	16.30 (2.74)	16.56 (2.61)	*16.40 (3.14)*	18.03 (2.77)	*18.32 (3.13)*
LVPWs/BSA (mm/m^2^)	6.97 (1.28)	5.81 (0.98)^*^		5.94 (1.41)^**^	
Adjusted Means	7.03 (1.23)	5.86 (1.18)	*6.02 (1.27)*	5.77 (1.24)	*5.74 (1.26)*
**Tissue Doppler mitral valve**
Septal é (cm/s)	12.47 (1.90)	11.50 (2.38)^*^		10.61 (1.40)^**^	
Adjusted means	12.47 (1.92)	11.57 (1.83)	*11.55 (2.10)*	10.55 (1.95)	*10.57 (2.10)*
Lateral é (cm/s)	18.13 (3.69)	14.88 (2.35)^**^		14.21 (2.23)^**^	
Adjusted means	18.17 (3.13)	14.97 (2.99)	*15.21 (2.41)*	14.05 (3.17)	*13.90 (2.40)*
Mean é (cm/s)	15.30 (2.35)	13.20 (2.01)^**^		12.41 (1.52)^**^	
Adjusted means	15.32 (2.08)	13.27 (1.99)	*13.37 (1.89)*	12.30 (2.11)	12.23(1.88)^b^
Septal ś (cm/s)	8.41 (1.13)	8.34 (1.11)		7.90 (1.26)	
Adjusted means	8.41 (1.21)	8.35 (1.15)	*8.37 (1.29)*	7.89 (1.22)	*7.87 (1.29)*
Lateral ś (cm/s)	10.27 (1.75)	8.80 (2.27)^**^		8.66 (2.08)^**^	
Adjusted means	10.26 (2.05)	8.79 (1.96)	*8.99 (2.35)*	8.70 (2.06)	*8.48 (2.35)*
Mean ś (cm/s)	9.34 (1.15)	8.57 (1.41)^*^		8.27 (1.28)^**^	
Adjusted means	9.34 (1.30)	8.57 (1.24)	*8.68 (1.44)*	8.30 (1.32)	*8.17 (1.44)*
**LV-function and mass**
FS (%)	39.98 (6.21)	38.23 (5.73)		34.29 (6.34)^***^	
Adjusted means	38.87 (6.30)	38.34 (6.01)	*38.02 (6.62)*	34.41 (6.39)	*34.32 (6.61)*
LVEF (%)	62.21 (3.84)	60.34 (3.00)		57.50 (5.03)^***^	
Adjusted means	62.08 (4.12)	60.39 (3.93)	*60.00 (4.26)*	57.72 (4.17)	*57.82 (4.24)*
LV-Mass/BSA (g/m^2^)	73.94 (18.87)	66.15 (13.29)		63.65 (15.38)	
Adjusted means	75.35 (16.22)	67.42 (15.05)^*^	*68.21 (14.30)*	63.58 (16.89)^*^	*61.66 (14.27)*

*BSA, body surface area; IVSd, septal diastolic diameter; IVSs, septal systolic diameter; LVIDd, left ventricular diastolic inner diameter; LVIDs, left ventricular systolic inner diameter; LVPWd, left ventricular posterior wall diastolic diameter; LVPWs, left ventricular posterior wall systolic diameter; SF, shortening fraction; LVEF, left ventricular ejection fraction calculated by Simpson method; LV-mass, left ventricular mass. Values are shown as mean (SD). Group differences were calculated by one-way analysis of variance adjusting for heartrate and height (ANCOVA). Intergroup differences were calculated by the Bonferroni post-hoc test with correction for multiple comparisons*;

* *p <0.05 vs. control*,

** *p <0.005 vs. control*,

*** *p <0.05 vs controls and LAD group*,

a *Subgroup analysis LAD vs. HAD group adjusted for follow-up time and radiotherapy—adjusted means (SD)*,

b *p <0.05 vs. LAD group. Italic values are subgroup analysis*.

Tissue Doppler mean septal and lateral wall systolic 's-wave was lower in both the CCS groups compared with controls (*p* = 0.002 for HAD group and *p* = 0.029 for LAD group). Mean tissue Doppler é-wave (as a measurement of diastolic function) was lower in the CCS groups compared with controls: (*p* = 0.001 for LAD group and *p* < 0.001 for HAD group). In the subgroup analysis comparing CCS groups, adjusting for follow-up time and radiotherapy mean é-wave was lower in the HAD group compared with the LAD group (*p* = 0.049).

### Correlations of Cardiovascular Outcome Variables Within the CCS Groups

**Figure 1:**
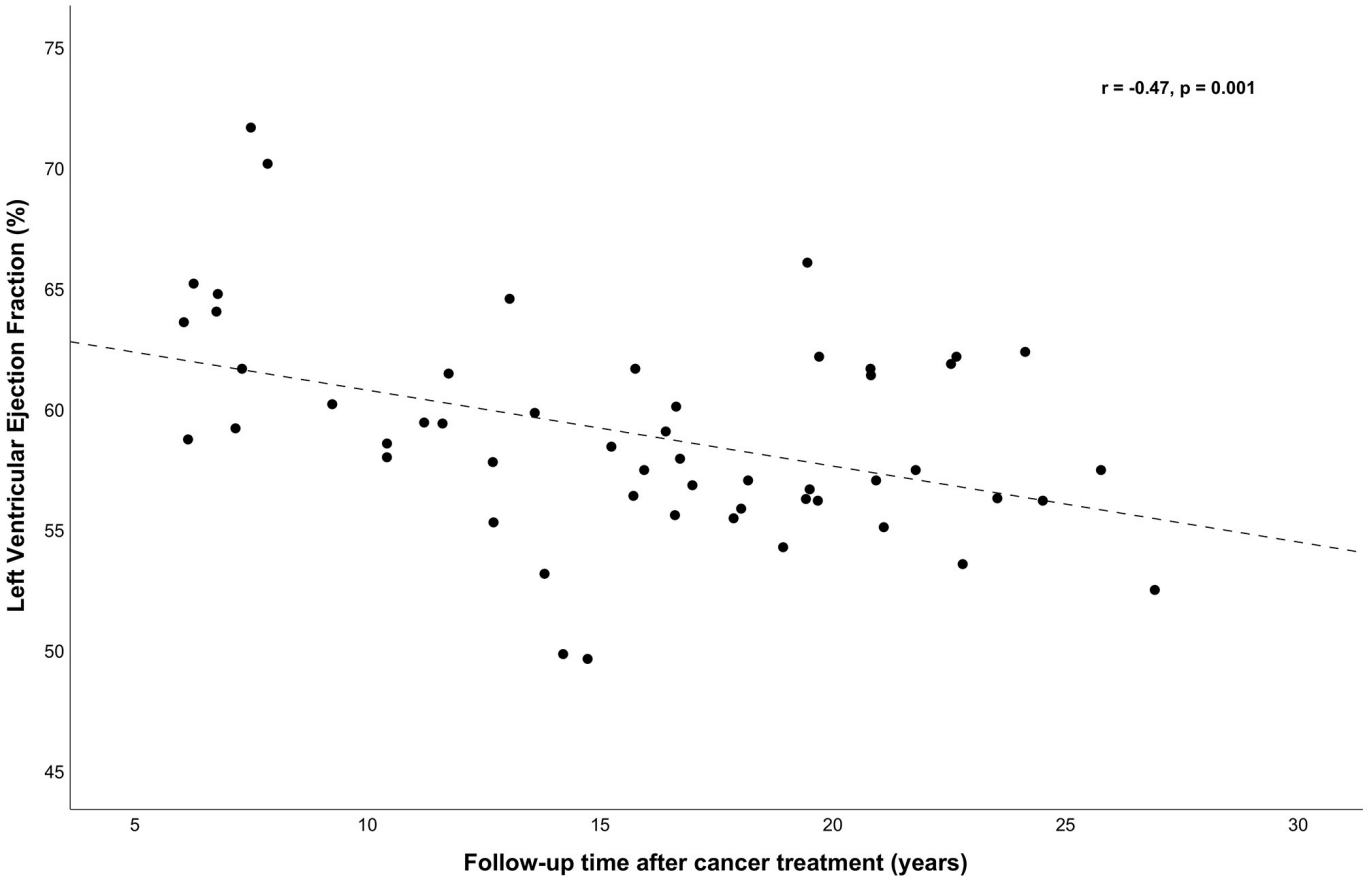
Left ventricular ejection fraction (LVEF, %) was correlated (adjusted for sex and age) with follow-up time (*r* = −0.47, *p* = 0.001). With simple linear regression a significant regression equation was found [*F*_(1, 51)_ = 10.65, *p* = 0.002], with an *r*^2^ = 0.173. The predicted LVEF (%) was 63.86–0.315 (years of follow-up time after cancer treatment).

Correlation analyses between total AC dose and outcome variables corrected for age and sex revealed moderate negative correlations with LVEF (*r* = −0.46, *p* < 0.001), SF (*r* = −0.43, *p* = 0.002), and with mean right and left CCA DI, a weak-moderate correlation (*r* = −0.30, *p* = 0.046). Cranial radiotherapy was correlated only with mean right and left CCA DI and SI (*r* = −0.31, *p* = 0.038 for DI and *r* = 0.31, *p* = 0.034 for SI). There were no significant correlations of CIMT, PAT-RHI and lipids and apolipoproteins regarding AC dose, cranial nor mediastinal radiotherapy and follow-up time (data not shown). Follow-up time after cancer treatment was associated with LVEF only (*r* = −0.47, *p* = 0.001, Figure 1).

**Figure 2:**
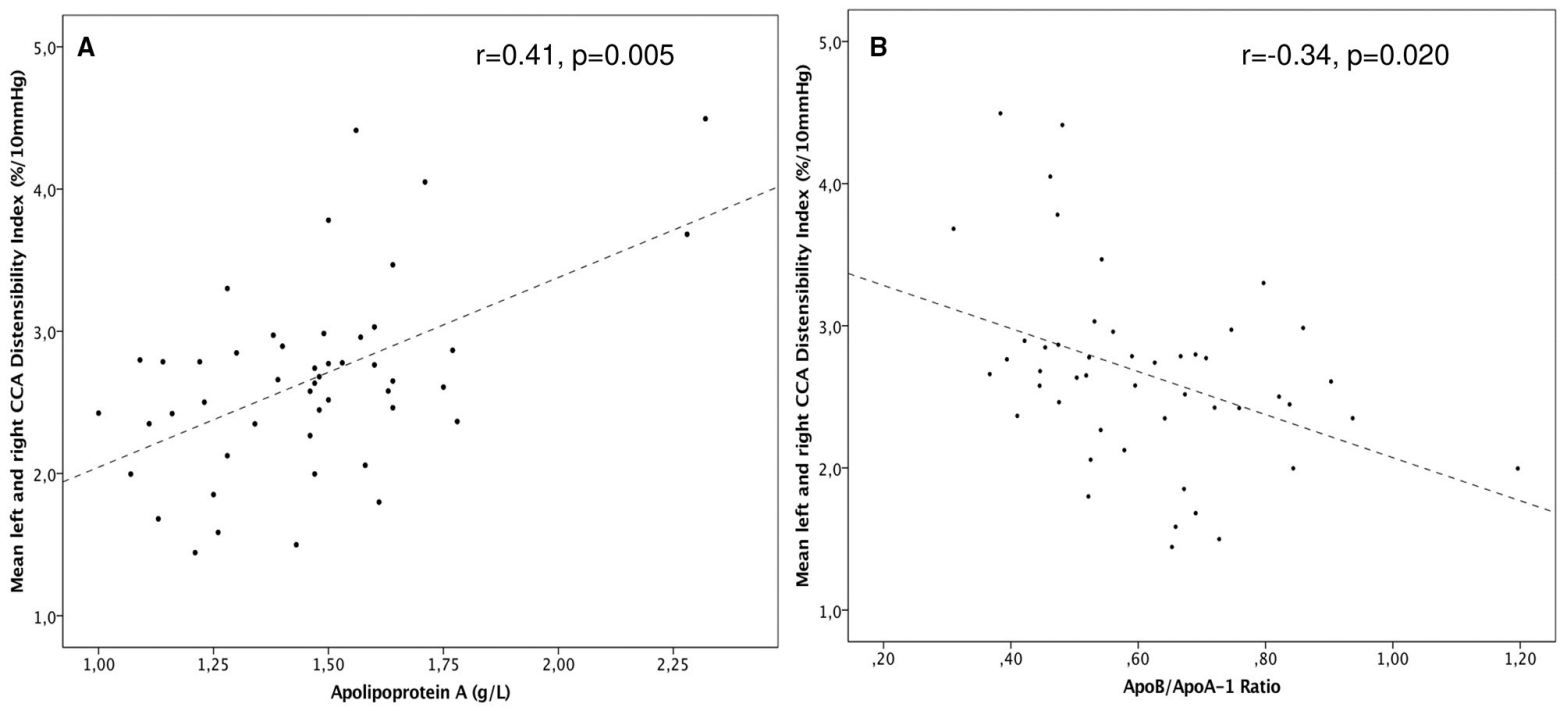
**(A)** Scatterplot of common carotid distensibility (CCA DI) among CCS was positively correlated with Apo-A (*r* = 0.41*, p* = 0.005). **(B)** DI vs. the Apo-B/Apo-A1-ratio among CCS. A higher ratio depicts lower DI (*r* = –0.34 *p* = 0.020).

When focusing on correlations between structural, functional, and biochemical outcome variables, mean right and left CCA DI and SI were correlated with Apo-A1 (*r* = 0.41, *p* = 0.005 for DI, Figure 2A and *r* = −0.32, *p* = 0.030 for SI), whereas Apo-B/Apo-A1 ratio met statistical significance only in correlation with DI (*r* = −0.34, *p* = 0.020, Figure 2B). PAT-RHI and CIMT did not correlate with any of the lipid or apolipoprotein markers.

## Discussion

Improvement of long-term follow up of CCS is mandatory to further lower the risk for cardiovascular events in adult life. The findings of this study demonstrate that subclinical cardiac, vascular, and lipid and apolipoprotein disorders are present in CCS already at a young adult age. More specifically, cardiac systolic and diastolic function and the carotid artery's elasticity are affected in CCS with a higher burden of cancer treatment, and the latter correlates with apolipoprotein abnormalities. These ultrasonographical and lipid and apolipoprotein markers are known risk-factors for future CVD in different patient populations (25–29), but how they predict CVD in CCS remains elusive.

### Carotid and Cardiac Markers

Carotid stiffness, a surrogate non-invasive marker for atherosclerosis, appears to be an independent marker for CVD and mortality (28, 29) and has been suggested to predict risk for stroke in older CCS (>40 years) (12). In the current study, CCS with a higher AC dose (HAD group) had the most increased carotid stiffness. Similar results were reported in a previous work of CCS (4), where the aortic stiffness was worse in CCS treated with AC as compared with healthy controls. Moreover, in a large study of 852 CCS by Arnold et al. (30), arterial SI was markedly higher than in healthy controls with more pronounced differences with increasing age even among those without hypertension. Carotid stiffness (expressed by DI and SI) in the current study correlated with cranial radiotherapy, indicating that both cranial radiotherapy and AC might account for the observed changes in carotid function. In young cancer survivors after childhood stem cell transplantation, radiotherapy was also reported to be associated with increased carotid stiffness and carotid plaques despite the use of protective shielding of the carotid area (31) suggesting a systemic effect. Radiotherapy in higher doses to the carotid area is known to cause atherosclerosis (12, 31).

In the present study, the subclinical changes in the cardiac function (both systolic and diastolic) and in carotid elasticity in CCS showed no correlation with each other. Theoretically, the increased afterload caused by impaired carotid elasticity could affect left ventricular diastolic parameters. This has been described in hypertensive patients in whom arterial stiffening was associated with left ventricular hypertrophy and impaired diastolic function (32). In young CCS the relationship between arterial stiffness and left ventricular diastolic dysfunction might be less obvious since AC and radiotherapy damage both vascular endothelium and cardiac myocytes and the “normal” timing of events could thus be disrupted. CCS are different from hypertensive patients, as that they do not have left ventricular hypertrophy but instead thinner cardiac walls and decreased left ventricular mass due to cardiomyocyte apoptosis (33). The expected alterations in left ventricular function due to stiffened vasculature in CCS could also require more sensitive methods such as strain rate imaging (34). The younger age of CCS in our study could be important too, since subtle left ventricular structural changes due to arterial stiffness may not be measurable in younger individuals (35).

These effects on the vasculature by cardiotoxic AC can be explained by the putative endothelial toxicity of AC through oxidative stress and direct DNA-damage acting on vascular endothelial cells, leading to apoptosis and endothelial dysfunction (36). It has also been reported that AC leads to decreased elastin and increased collagen content, suggesting a possible structural derangement caused by AC (37). Like AC, radiotherapy induces oxidative stress (38) leading to inflammation and fibrosis that acts locally in the irradiated area (39). But also, nearby or systemically through the so-called “non-targeted-effects” (40, 41) which could in part explain why cranial radiotherapy in the current study correlated with carotid elasticity. However, to separate the individual effects of AC and radiotherapy is complex. The HAD group had higher AC doses and cranial radiotherapy as well as a longer follow-up time and the effects are probably additive (42). There are also other factors in CCS such as previous infections and severity of disease that could affect the cardiovascular system as it has been reported that CCS without chemotherapy or radiotherapy have cardiovascular abnormalities when compared with controls (43). Furthermore, mediastinal radiotherapy in combination with AC increases the risk for CVD substantially (3, 44). We could not confirm this in the present study, probably due to relatively short follow-up time (45).

### Endothelial Function Assessed With PAT-RHI

In the present study, endothelial vasomotor function assessed with PAT-RHI was decreased in CCS but no correlation with carotid stiffness nor cardiac function was observed. PAT-RHI has been reported to correlate with endothelial vasomotor response in the coronary circulation (24) and to predict cardiovascular outcome in individuals with CVD risk factors (25, 46). PAT-RHI has been widely studied in other patient cohorts but to our knowledge only two previous studies assessed PAT-RHI in CCS: one in young acute lymphoblastic leukemia survivors (47) and the other in young Hodgkin lymphoma survivors (48). Interestingly, in our study, the LAD group, but not the HAD group, had lower PAT-RHI than controls. Mediastinal radiotherapy was more frequent in the LAD group in the current study while in patients with Hodgkin lymphoma in the previous study (48) mediastinal radiotherapy in conjunction with chemotherapy was associated with a lower PAT-RHI score. The underlying mechanism is unclear, but one explanation could reside in the systemic effects on the endothelial cells of radiotherapy and AC (38, 39). However, despite the growing data that PAT-RHI can predict cardiovascular morbidity, its potential benefit in general population remains debatable and results should be interpreted with caution (49).

### Apolipoproteins and Lipids

In the INTERHEART study of 12,461 individuals with myocardial infarction, an increased Apo-B/Apo-A1 ratio was shown to be superior to any lipid variable in estimating the risk for myocardial infarction across ethnic groups, sexes, and at all ages (26). Likewise, reported in that study, for every increase in Apo-A1 by 1 SD, cardiovascular risk decreased by 33% compared to 15% for a similar increase in HDL. In the present study, Apo-A1 and the Apo-B/Apo-A1 ratio among CCS were associated with better- and worse carotid elasticity respectively. Apo-A1, along with HDL, is well-known for its vasoprotective effects, being involved in the “reverse cholesterol transport” from the periphery to the liver and in arterial plaque inhibition in part *via* antiinflammatory effects (50). Apo-A1 is the major protein component of HDL and has been suggested to protect the cardiovascular system against AC toxicity *via* cytoprotective effects and cardiac “sparing” in delivery of AC (51). Whether Apo-A1 might be the driving mechanism of the effect observed herein needs to be assessed in future studies.

Apo-B, suggested to be the basic unit of injury to the arterial wall (52), was higher in the HAD group and in conjunction with this nearly 30% of CCS in the HAD group had Apo-B/Apo-A1-ratio over the upper reference limit, with lower number in the LAD group (15%) but still higher compared with controls (7%). This difference could be explained by cranial radiotherapy which was used in 31% of the HAD group compared to none in the LAD group. Cranial radiation, which was used only the HAD group, has been linked to neuroendocrine dysfunction and growth hormone deficiency that can cause dyslipidemia (14, 53, 54). Cranial radiotherapy was also correlated with impaired carotid elasticity, and this correlation could be due to the above-mentioned systemic effects of radiotherapy including inflammation and neuroendocrine dysfunction (14, 41, 53, 54). Importantly, normal-weight CCS had higher values of analysed apolipoprotein biomarkers. Based on this observation, we propose that lipid and apolipoprotein screening should be added to the current monitoring of CCS even in those without other signs of metabolic syndrome.

### Limitations

The cross-sectional design and the relatively small number of CCS is an important limitation. Given the cohort size, we did not use the three-group classification (in example: low risk <100 mg/m^2^, intermediate risk 100–250 mg/m^2^, and high risk >250 mg/m^2^) commonly used in cardiovascular follow-up of CCS (44). Moreover, we did not account for diet and measures of fat deposition, which would have been useful to further understand the lipid data. Additional biomarkers used in cardiovascular risk assessment such as blood glucose and brain natriuretic peptide would have better profiled the CCS cohort. We did not use cardiac strain or strain-rate which could have been useful in the assessments of associations between vascular and cardiac measures. Studies in this regard are ongoing at our institution.

### Strengths

The strengths of the study are; 1) the young CCS cohort, 2) absence of chronic diseases, 3) absence of ongoing medical treatments, and 4) the narrow age-span of the study cohort. The methods used are also widely available and reproducible and future follow up of the study cohort is possible.

## Conclusions

Young Swedish CCS without overt CVD show changes in the cardiovascular system, lipid, and apolipoprotein profiles with potential implications for their CVD risk later in life. Large prospective studies are needed to further assess the course and significance of these changes in clinical CVD.

## Data Availability Statement

The raw data supporting the conclusions of this article will be made available by the authors, without undue reservation.

## Ethics Statement

The studies involving human participants were reviewed and approved by Etiknämnden Lunds Universitet (DNR 2013/205). The patients/participants provided their written informed consent to participate in this study.

## Author Contributions

OB: study design, data collection, and manuscript writing. CW, TW, IØ, and PL: study design and manuscript writing. All authors contributed to the article and approved the submitted version.

## Funding

This study was funded by the Swedish Childhood Cancer Fund and the Swedish Heart-Lung Foundation (grant sp2017).

## Conflict of Interest

The authors declare that the research was conducted in the absence of any commercial or financial relationships that could be construed as a potential conflict of interest.

## Publisher's Note

All claims expressed in this article are solely those of the authors and do not necessarily represent those of their affiliated organizations, or those of the publisher, the editors and the reviewers. Any product that may be evaluated in this article, or claim that may be made by its manufacturer, is not guaranteed or endorsed by the publisher.

## Abbreviations

Apo-B: apolipoprotein B
Apo-A1: apolipoprotein A1
AC: anthracyclines
CCA: common carotid artery
CCS: childhood cancer survivors
CVD: cardiovascular disease
DI: distensibility index
HAD: high anthracycline dose
HDL: high density lipoprotein
IVS: interventricular septum
LAD: low anthracycline dose
LDL: low density lipoprotein
LVEF: left ventricular ejection fraction
LVID: left ventricular inner diameter
LVPW: left ventricular posterior wall
PAT-RHI: peripheral arterial tonometry-reactive hyperaemia index
SF: shortening fraction
SI: stiffness index
TG: triglycerides.
